# A network analysis of anxiety and depression symptoms among Chinese nurses in the late stage of the COVID-19 pandemic

**DOI:** 10.3389/fpubh.2022.996386

**Published:** 2022-11-02

**Authors:** Pu Peng, Qiongni Chen, Mining Liang, Yueheng Liu, Shubao Chen, Yunfei Wang, Qian Yang, Xin Wang, Manyun Li, Yingying Wang, Yuzhu Hao, Li He, Qianjin Wang, Junhong Zhang, Yuejiao Ma, Haoyu He, Yanan Zhou, Zejun Li, Huixue Xu, Jiang Long, Chang Qi, Yi-Yuan Tang, Yanhui Liao, Jinsong Tang, Qiuxia Wu, Tieqiao Liu

**Affiliations:** ^1^Department of Psychiatry and National Clinical Research Center for Mental Disorders, The Second Xiangya Hospital of Central South University, Changsha, China; ^2^Clinical Nursing Teaching and Research Section, The Second Xiangya Hospital, Central South University, Changsha, China; ^3^Department of Psychology, College of Education, Hunan First Normol University, Changsha, China; ^4^Department of Psychiatry, Hunan Brain Hospital (Hunan Second People's Hospital, Changsha, China; ^5^Shanghai Mental Health Center, Shanghai Jiao Tong University School of Medicine, Shanghai, China; ^6^Department of Psychiatry, Zhejiang Provincial People's Hospital, People's Hospital of Hangzhou Medical College, Hangzhou, China; ^7^College of Health Solutions, Arizona State University, Phoenix, AZ, United States; ^8^Department of Psychiatry, Sir Run Shaw Hospital, School of Medicine, Zhejiang University, Hangzhou, China

**Keywords:** depression, anxiety, network analysis, COVID-19 pandemic, nurse

## Abstract

**Background:**

Nurses are at high risk for depression and anxiety symptoms after the outbreak of the COVID-19 pandemic. We aimed to assess the network structure of anxiety and depression symptoms among Chinese nurses in the late stage of this pandemic.

**Method:**

A total of 6,183 nurses were recruited across China from Oct 2020 to Apr 2021 through snowball sampling. We used Patient Health Questionnaire-9 (PHQ-9) and Generalized Anxiety Disorder scale-7 (GAD-7) to assess depression and anxiety, respectively. We used the Ising model to estimate the network. The index “expected influence” and “bridge expected influence” were applied to determine the central symptoms and bridge symptoms of the anxiety-depression network. We tested the stability and accuracy of the network *via* the case-dropping procedure and non-parametric bootstrapping procedure.

**Result:**

The network had excellent stability and accuracy. Central symptoms included “restlessness”, “trouble relaxing”, “sad mood”, and “uncontrollable worry”. “Restlessness”, “nervous”, and “suicidal thoughts” served as bridge symptoms.

**Conclusion:**

Restlessness emerged as the strongest central and bridge symptom in the anxiety-depression network of nurses. Intervention on depression and anxiety symptoms in nurses should prioritize this symptom.

## Introduction

Approximately two-fifths of the nurses suffered from anxiety and depression symptoms after the breakout of the COVID-19 pandemic (1), which led to their impaired quality of life and attrition (2, 3). Moreover, growing evidence demonstrated the psychological harm of the pandemic persisted even long after the initial peak of the pandemic (4–8). These studies warranted long-term investigation, identification, and intervention of mental distress among nurses in the late stage of COVID-19.

Despite the high interest in the prevalence and correlation of mental distress among nurses, few studies assessed them at a symptom level. The use of total scores of a scale as an index of mental distress (e.g., calculating the total scores of depression measurement tools and interpreting it as the severity of depression) in previous studies failed to point out the difference and the interaction within the symptoms (9). In contrast, a symptom-based approach might provide more useful insights into the establishment, prevention, and treatment of mental distress (9). Network analysis is a promising tool for understanding the symptomology of psychiatric disorders (10). It assumes that disorders are composed of interacting symptoms, which permits finding the most influential symptoms (i.e., “central symptom”) in the disorders (11). It also provides new insights into the incidence of comorbidity by identifying the bridge symptoms across networks of different disorders (12). Center and bridge symptoms are vital in triggering and maintaining the disorder network, which might serve as potential intervention targets. Increasing studies applied the network approach to assess anxiety and depression among the general population, college students, adolescents, and clinicians during the COVID-19 pandemic, which documented both common and unique depression-anxiety network across different populations (13–17). However, as far as we know, no previous study described anxiety and depression symptoms among nurses via the network model.

Hence, we conducted the present study to assess anxiety and depression symptoms among a large sample of Chinese nurses with the network approach in the late stage of the COVID-19 pandemic. We aimed to demonstrate the central and bridge symptoms within the depression-anxiety network among nurses, which might be valuable in preventing and treating depression and anxiety in the nursing population.

## Materials and methods

### Study setting and participants

The online questionnaire-based cross-sectional study was conducted from October 2020 to April 2021. During this period, China entered the late stage of the COVID-19 pandemic. Despite the strict “Zero-COVID-19” policy (18), the nationwide lockdown policy was ended. Only two cities experienced a short local lockdown in this period. Participants were recruited with a snowball sampling technique. The online survey was conducted on Wenjuanxin (a popular questionnaire website in China). We distributed the survey link through WeChat, the most widely used social platform in China. Participants were encouraged to forward this survey link to their friends. The inclusion criteria were as follows: (1) aged over 18 and Chinese; (2) worked as nurses during the pandemic; and (3) willing to take participate in this study. Nursing students were not included. All participants gave informed consent. Participants could only submit the questionnaires after responding to all questions. The study was approved by the ethics committee of the Second Xiangya Hospital of Central South University.

### Measures

Demographic characteristics (age, gender, married status, and education level) and work-related characteristics (occupation, practicing years, title, and workplace) were collected.

Depression and anxiety were assessed *via* Patient Health Questionnaire-9 (PHQ-9) and General Anxiety Disorder scale-7 (GAD-7). Both of the scales applied a 4-Likert-like question and participants rated the frequency of anxiety and depression symptoms ranging from 0 (Not at all) to 3 (Almost every day). The two scales gained strong validity and were widely used in the Chinese population (19, 20). The Cronbach's alpha of PHQ-9 and GAD-7 in our study was 0.901 and 0.929, showing excellent reliability. A cutoff point of five was used to screen for depression and anxiety symptoms.

### Statistical analysis

To describe the data, we presented the continuous data as the median and interquartile range (IRQ; 25–75%) and the categorical data as frequency and percentage. All the tests were 2-tailed, and p < 0.05 suggested statistically significant. All the statistical analysis was done in R (ver.4.2.0).

### Network estimation

We calculated the means and standard deviations (SD) of items in PHQ-9 and GAD-7 *via* the R package “psych”. Items were excluded when the SD was 2.5 times lower than the mean value of the items (21). The function “goldbricker” in the R package “networktools” was used to screen for redundant items.

The distribution of the PHQ-9 and GAD-7 scores was positively skewed. Following previous studies (22, 23), we binarized each item value to the absence (item recorded as 0) and presence (item recorded as 1 or 2 or 3). We applied the Ising model to estimate the depression-anxiety network, which was a popular method for constructing network from psychological binary data (24). It has been widely used in describing the network of depressive and/or anxiety symptoms in different populations (22, 25–27). In the Ising network, the nodes represent PHQ9 and GAD7 items, while the edges represent the independent association between item pairs (i.e., logistic regression coefficients after controlling for the rest variables in the network). To avoid spurious, false-positive edges, the gamma was set at 0.25, which allowed us to retain the most important associations between the variables within the network. The R package “qgraph”, “bootnet”, and “IsingFit” were used to estimate and visualize the network model. Blue edges indicate positive association. Thicker edges represented stronger association.

We calculated the centrality index “expected influence” (EI) to identify the importance of the symptoms in the network (28). Nodes with the highest EI were identified as the central symptoms. We also assessed the predictability of each symptom in the network through the R package “MGM”. Node with high predictability was considered to be easily influenced by its adjacent nodes. Targeting related nodes might be useful to control nodes with high predictability (14). We calculated the bridge expected influence (BEI) index to identify the bridge symptoms of the anxiety-depression network *via* the R package “networktools”. Bridge symptoms were chosen with an 80th percentile BEI threshold (15, 21).

### Network accuracy and stability

We employed non-parametric bootstrapping with 1,000 bootstrap samples through the “bootnet” packages to assess the accuracy of the edge. The case-dropping bootstrap approach was applied to test the stability of BEI and EI (29). The correlation stability coefficient (CS-C) represented the stability of the network. A CS-C higher than 0.5 was considered to be good.

## Result

### Study sample

Six thousand two hundred eighty Nurses Took Part in the Survey. After Removing Duplicates and Logical Errors (Such as Practicing Year Exceeding age and Wrong Answer in the Trap Question), 6,183 Participants Were Included in the Final Analysis (Table 1). The Median age Was 30 (25, 30), and the Median Practicing Year Was 8 (4, 14) Years. The Majority of the Participants Were Female (6,023, 97%), Had a Bachelor's Degree (4,129.67%), Had a Junior Title (3,764, 60%), and Were Married (4,175, 68%). The Prevalence of Depression and Anxiety Symptoms in Our Participants Was 58 and 54%, Respectively.

**Table 1:** Sample characteristics.

**Characteristic**	***N* = 6,183^1^**
**Age, year**	30 (26, 36)
**Gender**	
Female	6,023 (97%)
Male	160 (2.6%)
**Education level**	
Junior college or below	1,834 (30%)
Bachelor degree	4,129 (67%)
Master degree or above	220 (3.6%)
**Partnership**	
Single	1,202 (19%)
Partnered	612 (9.9%)
Married	4,175 (68%)
Widowed or divorced	194 (3.1%)
**Workplace**	
Tertiary hospital	2,397 (39%)
Secondary hospital	1,451 (23%)
Primary hospital	2,335 (38%)
Practicing year, year	8 (4, 14)
**Title**	
Junior title	3,734 (60%)
Nurse in charge	2,109 (34%)
Chief nurse	340 (5.5%)
**GAD7 scores**	5 (2, 8)
**PHQ9 scores**	5 (2, 8)
**Anxiety symptom**	3,366 (54%)
**Depression symptom**	3,564 (58%)

^1^Median (IQR); *n* (%).

### The network structure of anxiety-depression symptom

**Figure 1:**
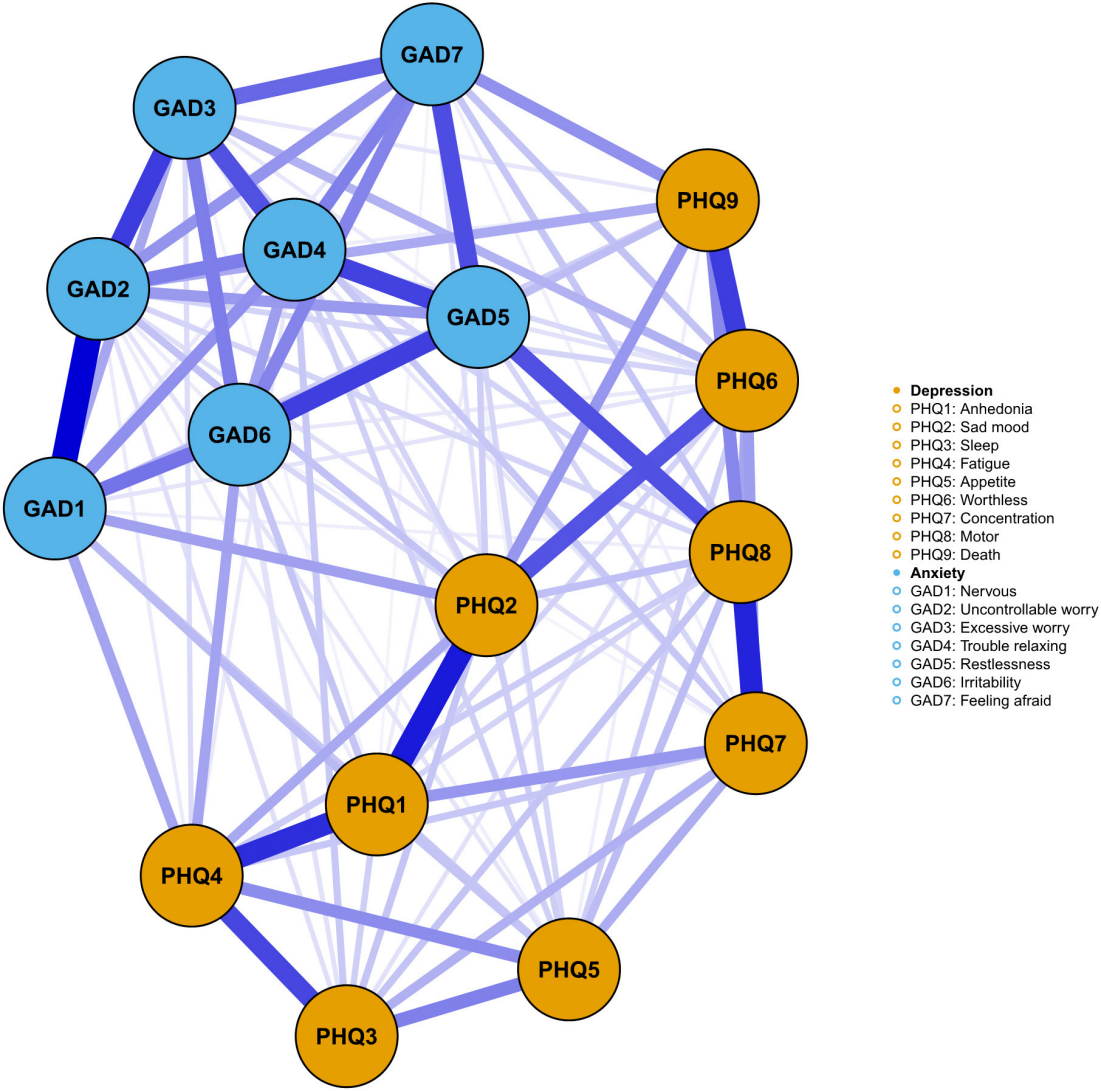
The network of anxiety-depression symptoms. Orange nodes represented the depression symptoms, while blue nodes represented the anxiety symptoms. The thickness of the edges represented the strength of the association between two nodes, with higher thickness indicating stronger relationship.

No items were excluded for redundancy and low informativeness (Table 2). Figure 1 showed the network structure of anxiety and depression. The density of the network was high (0.88, 106/120), with a mean weight of 0.428. All edges were positive. The strongest edge within the anxiety symptoms was the edge GAD1 (Nervous)—GAD2 (Uncontrollable worry), followed by GAD2 (Uncontrollable worry)—GAD3 (Excessive worry). The strongest edge within the depression symptoms was the edge PHQ1 (Anhedonia)—PHQ2 (Sad mood). PHQ8 (Motor) and GAD5 (Restlessness) showed the strongest association between anxiety and depression symptoms, which was statistically stronger than most of the other edges according to the nonparametric bootstrapping (Figure S1). Table S1 summarized the strength of each edge.

**Table 2:** Descriptive statistics of the PHQ-9 and GAD-7 items.

**Items**	**Item content**	**Mean**	**SD**	**Presence^1^**	**Absence^2^**	**Expected influence^3^**	**Predictability**
PHQ1	Anhedonia	0.95	0.74	4,535 (73%)	1,648 (27%)	−0.55	0.50
PHQ2	Sad mood	0.86	0.69	4,223 (68%)	1,960 (32%)	0.86	0.56
PHQ3	Sleep	1.10	0.89	4,446 (72%)	1,737 (28%)	−2.07	0.38
PHQ4	Fatigue	1.15	0.80	4,884 (79%)	1,299 (21%)	−0.11	0.47
PHQ5	Appetite	0.80	0.80	3,632 (59%)	2,551 (41%)	−1.76	0.47
PHQ6	Worthless	0.72	0.79	3,285 (53%)	2,898 (47%)	−0.05	0.61
PHQ7	Concentration	0.70	0.79	3,076 (50%)	3,107 (50%)	−0.80	0.60
PHQ8	Motor	0.44	0.67	2,154 (35%)	4,029 (65%)	0.37	0.53
PHQ9	Death	0.28	0.56	1,365 (22%)	4,818 (78%)	−0.73	0.26
GAD1	Nervous	0.88	0.74	4,082 (66%)	2,101 (34%)	0.74	0.61
GAD2	Uncontrollable worry	0.69	0.78	3,115 (50%)	3,068 (50%)	1.34	0.72
GAD3	Excessive worry	0.91	0.80	4,059 (66%)	2,124 (34%)	0.25	0.59
GAD4	Trouble relaxing	0.75	0.78	3,484 (50%)	2,699 (50%)	0.95	0.69
GAD5	Restlessness	0.44	0.65	2,237 (36%	3,946 (64%)	1.39	0.61
GAD6	Irritability	0.92	0.79	4,166 (67%)	2,017 (33%)	0.08	0.54
GAD7	Feeling afraid	0.51	0.70	2,501 (40%)	3,682 (60%)	0.08	0.59

^1^*n* (%); Item Recorded as 1 or 2 or 3 was considered to be “presence”.

^2^*n* (%); Item recorded as 0 was considered to be “absence”.

^3^Raw data from the depression-anxiety network.

### Central symptoms and bridge symptoms

**Figure 2:**
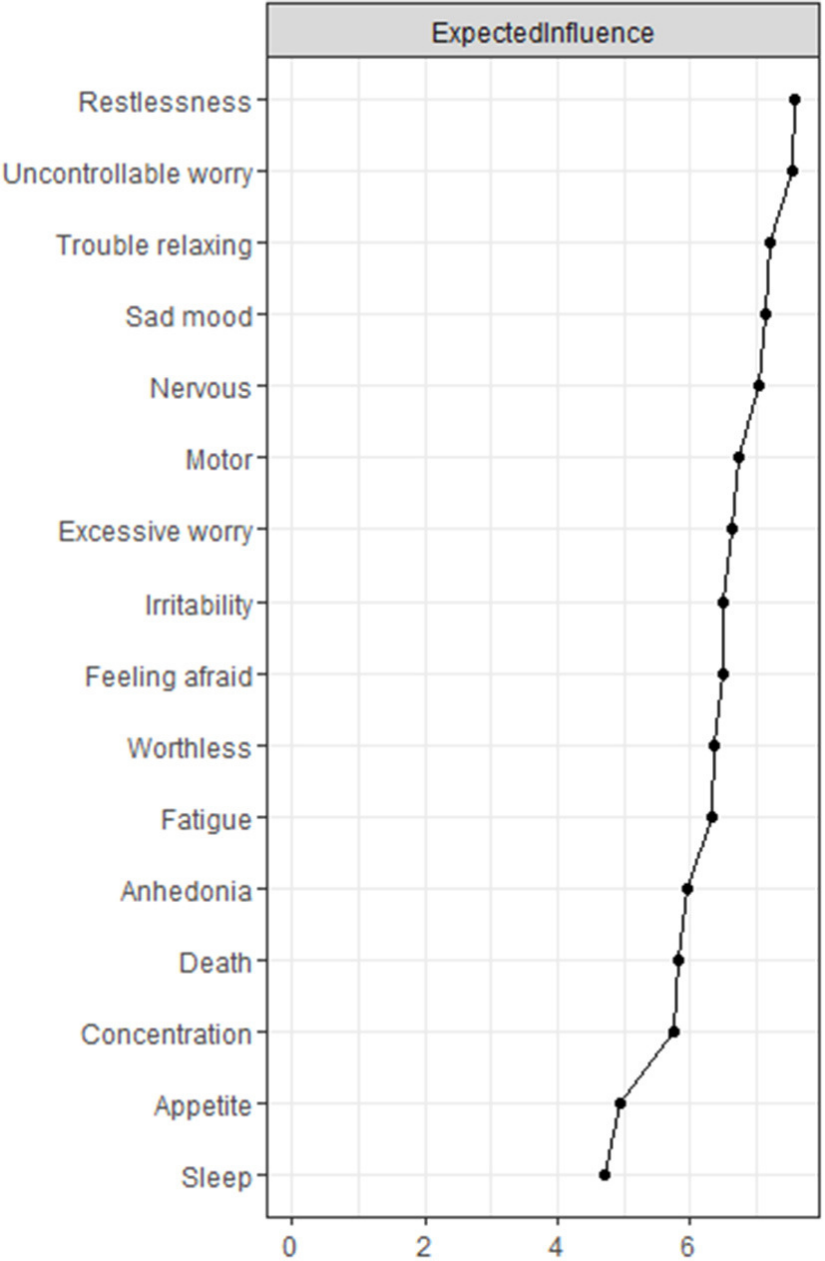
The node expected influence plot. The X-rays represented the expected influence of each node. Nodes with higher expected influence have stronger impact in other nodes within the network.

The centrality plot (Figure 2) suggested that GAD5 (Restlessness), GAD2 (Uncontrollable worry), and GAD4 (Trouble relaxing) were the central symptoms with the highest EI. The centrality differs test (Figure S2) showed these nodes were statistically stronger than other nodes in the network. In the depression community, PHQ2 (Sad mood) was the most influential symptom. PHQ3 (Sleep) and PHQ5 (Appetite) held the lowest EI, suggesting they might be marginal symptoms. The mean predictability was 0.55, implying that half of the variation might be explained by its neighbors (Table 2). The predictability of GAD2 (Uncontrollable worry) was highest (0.72), while PHQ9 (Death) held the lowest predictability (0.26).

**Figure 3:**
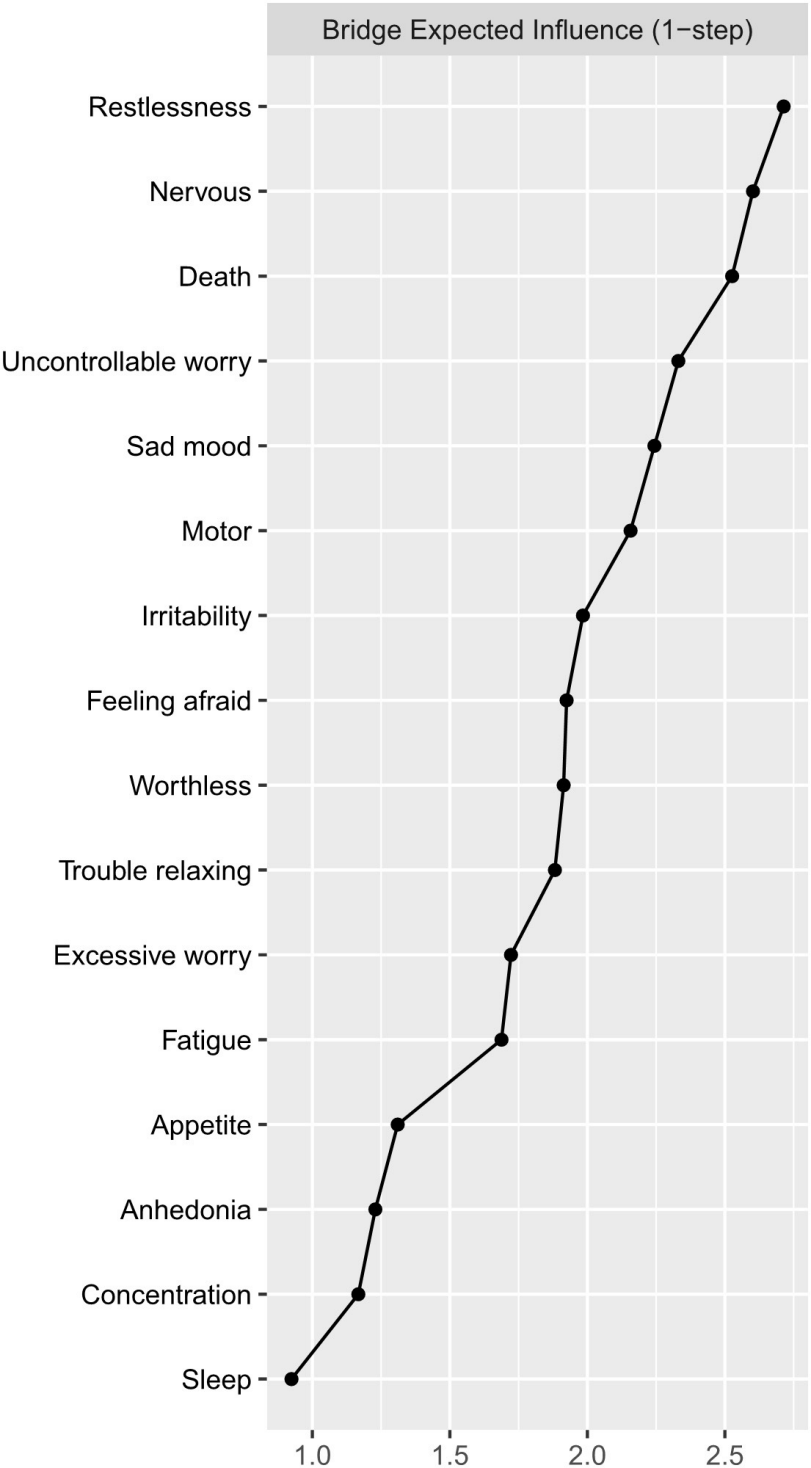
The bridge expected influence plot. The X-rays represented the bridge expected influence of each node. Nodes with higher bridge expected influence are recognized as bridge symptoms that drove the comorbid depression and anxiety symptoms.

The bridge expected influence index (Figure 3) indicated that GAD5 (Restlessness), GAD1 (Nervous), and PHQ9 (Death) were the bridge symptoms that drove the comorbid depression and anxiety symptoms. The correlation matrix of PHQ9 and GAD7 items were presented in Table S1. The strongest association between anxiety symptoms and depression symptoms lies in GAD5 (Restlessness) and PHQ8 (Motor), followed by GAD7 (Feeling afraid) and PHQ9 (Death), and GAD1 (Nervous) and PHQ2 (Sad mood).

### Network stability and accuracy

**Figure 4:**
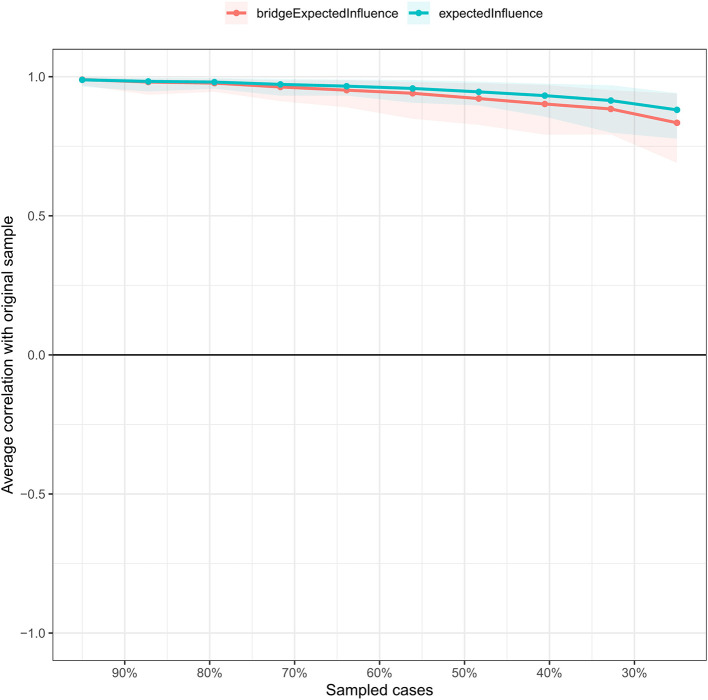
The stability of the depression-anxiety network. The stability of central and bridge expected influence by case-dropping bootstrap. The CS-C for the node and bridge expected influence was 0.672 and 0.75, respectively.

**Figure 5:**
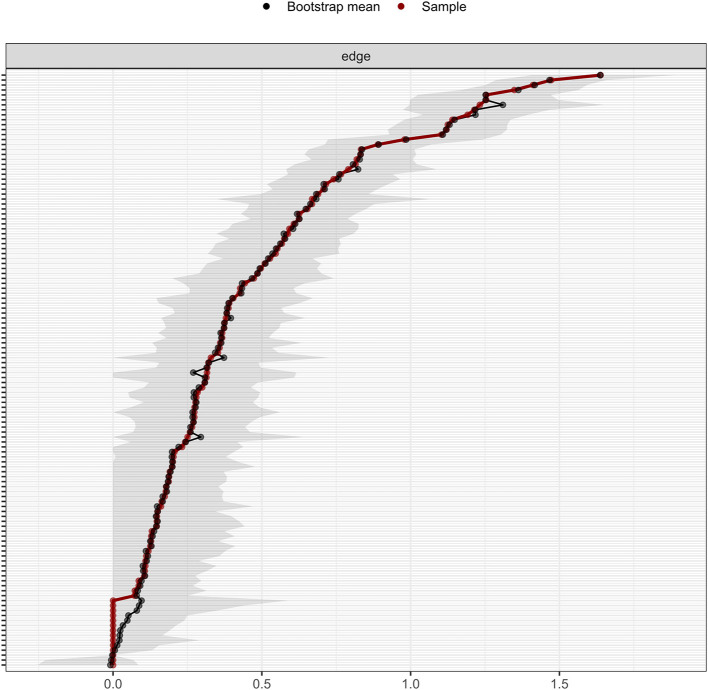
The accuracy of the network edges by non-parametric bootstrapping. The gray area represents the bootstrap 95% confidence interval.

The anxiety-depression network showed excellent stability. The CS-C of node EI was 0.75, implying the EI was still correlated with the original data (*r* = 0.75) after dropping out 75% of the data (Figure 4). The CS-C of BEI was 0.672, which was also very good. Figure 5 showed the result of the nonparametric bootstrap procedure. The bootstrapped 95% CIs were narrow, indicating high accuracy.

## Discussion

To our knowledge, it is the first study to describe the network of anxiety and depression symptoms among nurses in the late stage of the COVID-19 pandemic. The central symptom of the anxiety-depression network was restlessness, uncontrollable worry, trouble relaxing, and sad mood. Restlessness, nervous, and suicidal thoughts served as bridge symptoms linking anxiety and depression.

We found that GAD5 (Restlessness) was the most central symptom in the anxiety-depression network. It was positively associated with all anxiety symptoms and depression symptoms except PHQ4 (Fatigue) and PHQ3 (Sleep). Besides, other psychomotor symptoms such as GAD4 (Trouble relaxing) and PHQ8 (Motor) also displayed high EI. The high influence of psychomotor symptoms in the anxiety-depression network was consistently reported by numerous studies among the different populations across the world during the pandemic (16, 25, 31–34), which might result from the restriction on social and recreational activities due to the COVID-19 pandemic. Previous studies found psychomotor symptoms emerged as central symptoms during the peak of the pandemic when there was a strict motivation restriction, and showed remission when the restriction was relaxed (31). A longitudinal study also indicated psychomotor symptoms served as central symptoms during the pandemic period rather than the pre-pandemic period (32). Although the nationwide lockdown was ended in China when we conducted this study, nurses might decrease outdoor activities due to the busy work and the social distancing policy, which led to the high centrality of psychomotor symptoms. Similar findings were reported in Chinese clinicians during the late stage of the pandemic, suggesting the psychomotor symptoms might be the hallmark of the depression and anxiety symptoms of healthcare workers in this period, which warranted further attention (17).

GAD5 (Restlessness) was also the bridge symptom in this population, which implied that targeting restlessness was of great clinical value in treating both depression and anxiety disorders. The inter-connection between GAD5 (Restlessness) and PHQ8 (Motor) was the most robust transdiagnostic edge within the network of depression and anxiety symptoms. The transdiagnostic characteristic of restlessness was validated in both clinical (30) and community samples (16, 31, 33, 35) via the network model in previous studies. A longitudinal study provided more direct evidence, which found restlessness predicted subsequent relapse of major depressive disorder (MDD) (36). Also, early improvement of restlessness symptoms was positively associated with remission of MDD, suggesting the potential effectiveness of targeting restlessness in treating MDD (37).

PHQ2 (Sad mood) and GAD2 (Uncontrollable worry) were also central symptoms in the network. This finding supported the current status of the two symptoms as core and necessary symptoms required for the diagnosis of generalized anxiety disorder (GAD) and MDD (38, 39). Similar results were reported in different populations both before and after the breakout of the pandemic (15, 30, 40–42). In addition to restlessness, GAD1 (Nervous) showed high BEI, implying itself as a bridge symptom. Surprisingly, PHQ9 (Death) was another bridge symptom within the network, which has not been reported in previous studies. It exhibited a positive relationship with all anxiety symptoms and was tightly associated with GAD2 (Uncontrollable worry), GAD5 (Restlessness), and GAD7 (Feeling afraid). Further studies are in need to identify the relationship between anxiety and suicidal thoughts at a symptom level.

Interestingly, our study demonstrated the prominence of anxiety symptoms within the depression-anxiety network. The most central symptoms and bridge symptoms belonged to the anxiety communities, indicating that anxiety symptoms were vital in triggering and maintaining the depression-anxiety network among nurses. The fear and uncertainty of the recurrent emergence of the pandemic might account for this phenomenon (43, 44). Taken together, our results indicated the need for monitoring and intervening in several specific anxiety symptoms in the nursing population.

Our study had several clinical implications. First, the prevalence of depression (58%) and anxiety (54%) symptoms were high in the late stage of the pandemic. Timely screening and mental health intervention are in need. Second, we found restlessness served as the most central symptom and bridge symptoms across the anxiety-depression network. Our findings highlighted the priority of treating restlessness and related psychomotor symptoms in nurses. Targeted intervention on psychomotor symptoms such as improving physical activities and mindfulness-based approaches might be helpful in this population (30).

## Limitation

There were several limitations in our study. First, the cross-sectional study design didn't permit causal inferences. Second, we didn't assess the depression-anxiety network of our participants before the pandemic. Several important COVID-19 related information such as the history of COVID-19 infection, vaccine, and frontline experience were not collected. Hence, we could not directly evaluate the impact of the pandemic on depression and anxiety at a symptom level. Third, despite the large sample size, the use of convenience sampling might influence the representativeness of our samples. The majority of the participants were female, which might impact the generalizability of our results. Fourth, we used a self-administrated questionnaire rather than a clinical diagnostic interview. Fifth, depression and anxiety symptoms are highly associated with substance usage and medical or psychiatric illness, which we did not collect in the present study. Further studies are in need to determine how these factors impact the depression-anxiety network in the nursing population, which might provide valuable insights into the prevention and treatment of anxiety and depression in nurses.

## Conclusion

In conclusion, our study assessed the network structure of anxiety and depression symptoms among a large sample of nurses in the late stage of the COVID-19 pandemic. Restlessness emerged as both the strongest central symptom and bridge symptom in this network. Other central symptoms included uncontrollable worry, trouble relaxing, and sad mood. These key symptoms, especially psychomotor symptoms might hold great promise in the prevention and treatment of anxiety and depression in the nursing population.

## Data availability statement

The raw data supporting the conclusions of this article will be made available by the authors, without undue reservation.

## Ethics statement

The studies involving human participants were reviewed and approved by the Ethics Committee of the Second Xiangya Hospital of Central South University. The patients/participants provided their written informed consent to participate in this study.

## Author contributions

TL and QWu contributed to all aspects of the study. YLia, JT, JL, and CQ contributed to the study design. QY, PP, XW, and YH contributed to the statistical analysis. PP, YiW, MLi, and YM contributed to the drafting of the original manuscript. QC, YLiu, SC, MLia, ZL, HX, LH, and QWa contributed to the survey development and data acquisition. All authors contributed to the article and approved the submitted version.

## Funding

This study was supported by the Natural Science Foundation of Hunan Province (Grant No. 2020JJ4795 to TL).

## Conflict of interest

The authors declare that the research was conducted in the absence of any commercial or financial relationships that could be construed as a potential conflict of interest.

## Publisher's note

All claims expressed in this article are solely those of the authors and do not necessarily represent those of their affiliated organizations, or those of the publisher, the editors and the reviewers. Any product that may be evaluated in this article, or claim that may be made by its manufacturer, is not guaranteed or endorsed by the publisher.
