# Supplementary material for: A network analysis of anxiety and depression symptoms among Chinese nurses in the late stage of the COVID-19 pandemic
Source: Front Public Health. 2022 Nov 2;10:996386. doi: 10.3389/fpubh.2022.996386 (PMC9667894; doi:10.3389/fpubh.2022.996386)
Supplement: Supplementary file 1 [file Table_1.DOCX]

**Supplementary materials for “A network analysis of anxiety and depression symptoms among Chinese nurses in the late stage of the COVID-19 pandemic”**

**Supplementary Table 1** Correlation matrix of PHQ9 and GAD7 items

**Supplementary Figure 1** Estimation of edge difference within the depression-anxiety network by bootstrapped difference test

**Supplementary Figure 2** Estimation of node expected difference within the depression-anxiety network by bootstrapped difference test

**Supplementary Table 1** Correlation matrix of PHQ9 and GAD7 items

|  | PHQ1 | PHQ2 | PHQ3 | PHQ4 | PHQ5 | PHQ6 | PHQ7 | PHQ8 | PHQ9 | GAD1 | GAD2 | GAD3 | GAD4 | GAD5 | GAD6 | GAD7 |
| --- | --- | --- | --- | --- | --- | --- | --- | --- | --- | --- | --- | --- | --- | --- | --- | --- |
| PHQ1 | 0.000 | 1.472 | 0.308 | 1.348 | 0.382 | 0.280 | 0.669 | 0.273 | 0.000 | 0.443 | 0.128 | 0.086 | 0.000 | 0.125 | 0.374 | 0.073 |
| PHQ2 | 1.472 | 0.000 | 0.373 | 0.552 | 0.317 | 1.104 | 0.000 | 0.404 | 0.669 | 0.608 | 0.390 | 0.201 | 0.274 | 0.314 | 0.258 | 0.199 |
| PHQ3 | 0.308 | 0.373 | 0.000 | 1.191 | 0.819 | 0.191 | 0.510 | 0.379 | 0.000 | 0.000 | 0.206 | 0.188 | 0.353 | 0.000 | 0.177 | 0.000 |
| PHQ4 | 1.348 | 0.552 | 1.191 | 0.000 | 0.741 | 0.112 | 0.360 | 0.329 | 0.000 | 0.588 | 0.128 | 0.232 | 0.147 | 0.000 | 0.592 | 0.000 |
| PHQ5 | 0.382 | 0.317 | 0.819 | 0.741 | 0.000 | 0.320 | 0.527 | 0.425 | 0.107 | 0.367 | 0.178 | 0.000 | 0.300 | 0.270 | 0.109 | 0.086 |
| PHQ6 | 0.280 | 1.104 | 0.191 | 0.112 | 0.320 | 0.000 | 0.625 | 0.575 | 1.254 | 0.173 | 0.277 | 0.496 | 0.243 | 0.187 | 0.150 | 0.388 |
| PHQ7 | 0.669 | 0.000 | 0.510 | 0.360 | 0.527 | 0.625 | 0.000 | 1.411 | 0.485 | 0.000 | 0.203 | 0.358 | 0.164 | 0.265 | 0.072 | 0.105 |
| PHQ8 | 0.273 | 0.404 | 0.379 | 0.329 | 0.425 | 0.575 | 1.411 | 0.000 | 0.766 | 0.108 | 0.273 | 0.000 | 0.284 | 1.125 | 0.000 | 0.366 |
| PHQ9 | 0.000 | 0.669 | 0.000 | 0.000 | 0.107 | 1.254 | 0.485 | 0.766 | 0.000 | 0.316 | 0.546 | 0.161 | 0.119 | 0.427 | 0.250 | 0.707 |
| GAD1 | 0.443 | 0.608 | 0.000 | 0.588 | 0.367 | 0.173 | 0.000 | 0.108 | 0.316 | 0.000 | 1.638 | 0.567 | 0.715 | 0.473 | 0.890 | 0.147 |
| GAD2 | 0.128 | 0.390 | 0.206 | 0.128 | 0.178 | 0.277 | 0.203 | 0.273 | 0.546 | 1.638 | 0.000 | 1.254 | 0.832 | 0.653 | 0.130 | 0.689 |
| GAD3 | 0.086 | 0.201 | 0.188 | 0.232 | 0.000 | 0.496 | 0.358 | 0.000 | 0.161 | 0.567 | 1.254 | 0.000 | 1.121 | 0.147 | 0.833 | 0.979 |
| GAD4 | 0.000 | 0.274 | 0.353 | 0.147 | 0.300 | 0.243 | 0.164 | 0.284 | 0.119 | 0.715 | 0.832 | 1.121 | 0.000 | 1.214 | 0.625 | 0.815 |
| GAD5 | 0.125 | 0.314 | 0.000 | 0.000 | 0.270 | 0.187 | 0.265 | 1.125 | 0.427 | 0.473 | 0.653 | 0.147 | 1.214 | 0.000 | 1.232 | 1.141 |
| GAD6 | 0.374 | 0.258 | 0.177 | 0.592 | 0.109 | 0.150 | 0.072 | 0.000 | 0.250 | 0.890 | 0.130 | 0.833 | 0.625 | 1.232 | 0.000 | 0.791 |
| GAD7 | 0.073 | 0.199 | 0.000 | 0.000 | 0.086 | 0.388 | 0.105 | 0.366 | 0.707 | 0.147 | 0.689 | 0.979 | 0.815 | 1.141 | 0.791 | 0.000 |

PHQ1- Anhedonia, PHQ2-Sad mood, PHQ3-Sleep, PHQ4-Fatigue, PHQ5- Appetite, PHQ6- Worthless, PHQ7-Concentration, PHQ8-Motor, PHQ9-Death

GAD1- Nervous, GAD2-Uncontrollable worry, GAD3- Excessive worry, GAD4- Trouble relaxing, GAD5- Restlessness, GAD6- Irritability, GAD7- Feeling afraid

**Supplementary Figure 1** Estimation of edge difference within the depression-anxiety network by bootstrapped difference test. PHQ1- Anhedonia, PHQ2-Sad mood, PHQ3-Sleep, PHQ4-Fatigue, PHQ5- Appetite, PHQ6- Worthless, PHQ7-Concentration, PHQ8-Motor, PHQ9-Death, GAD1- Nervous, GAD2-Uncontrollable worry, GAD3- Excessive worry, GAD4- Trouble relaxing, GAD5- Restlessness, GAD6- Irritability, GAD7- Feeling afraid. Gray boxes indicate edges that do not significantly differ from one another. Black boxes represent edges that differ significantly from one another (α = 0.05).

**Supplementary Figure 2** Estimation of node expected difference within the depression-anxiety network by bootstrapped difference test. PHQ1- Anhedonia, PHQ2-Sad mood, PHQ3-Sleep, PHQ4-Fatigue, PHQ5- Appetite, PHQ6- Worthless, PHQ7-Concentration, PHQ8-Motor, PHQ9-Death, GAD1- Nervous, GAD2-Uncontrollable worry, GAD3- Excessive worry, GAD4- Trouble relaxing, GAD5- Restlessness, GAD6- Irritability, GAD7- Feeling afraid. Gray boxes indicate nodes that do not significantly differ from one another. Black boxes represent nodes that differ significantly from one another (α = 0.05).
