# Supplementary figures and images for: A network analysis of anxiety and depression symptoms among Chinese nurses in the late stage of the COVID-19 pandemic
Source: Front Public Health. 2022 Nov 2;10:996386. doi: 10.3389/fpubh.2022.996386 (PMC9667894; doi:10.3389/fpubh.2022.996386)

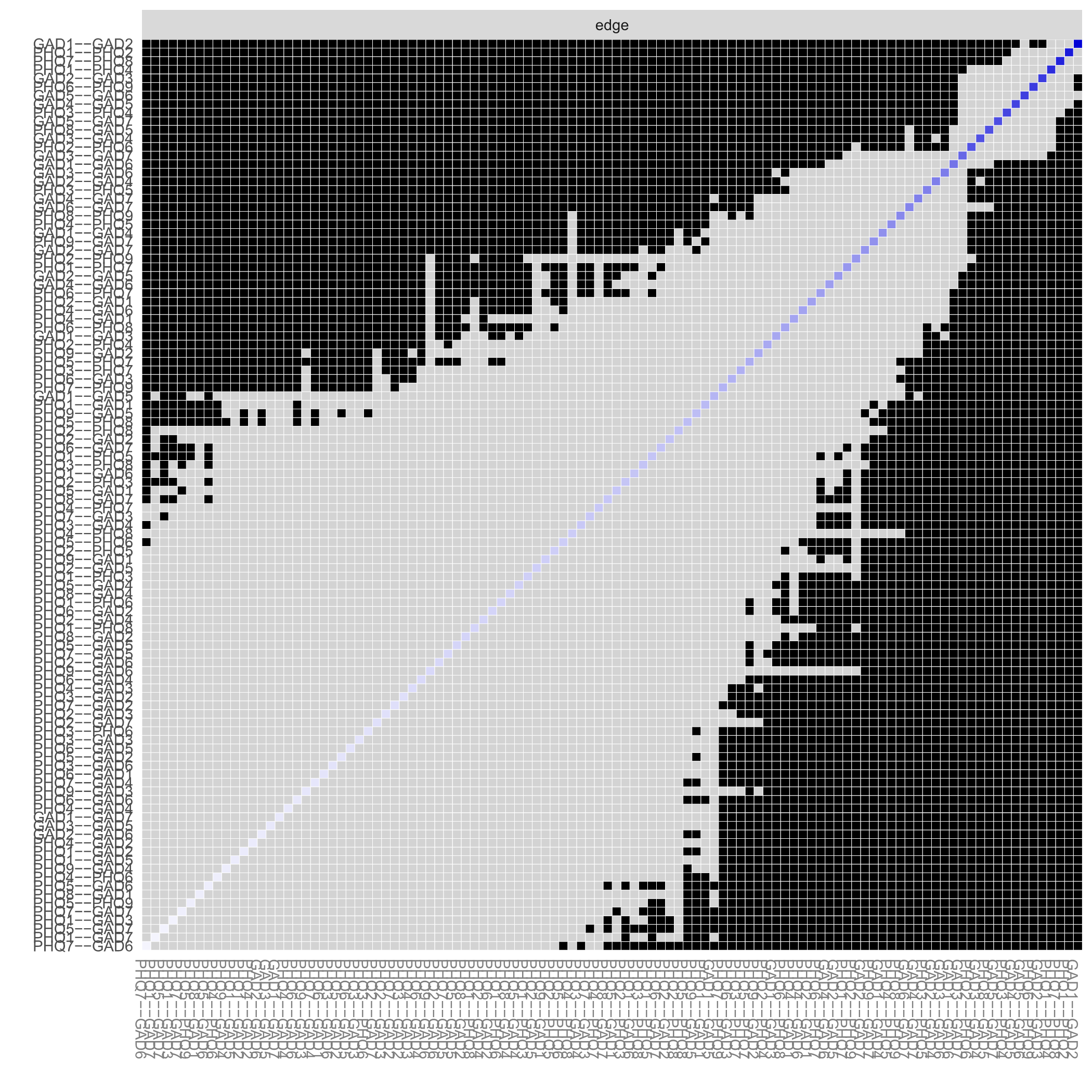

Supplement: Supplementary file 2 [file Image_1.TIFF]

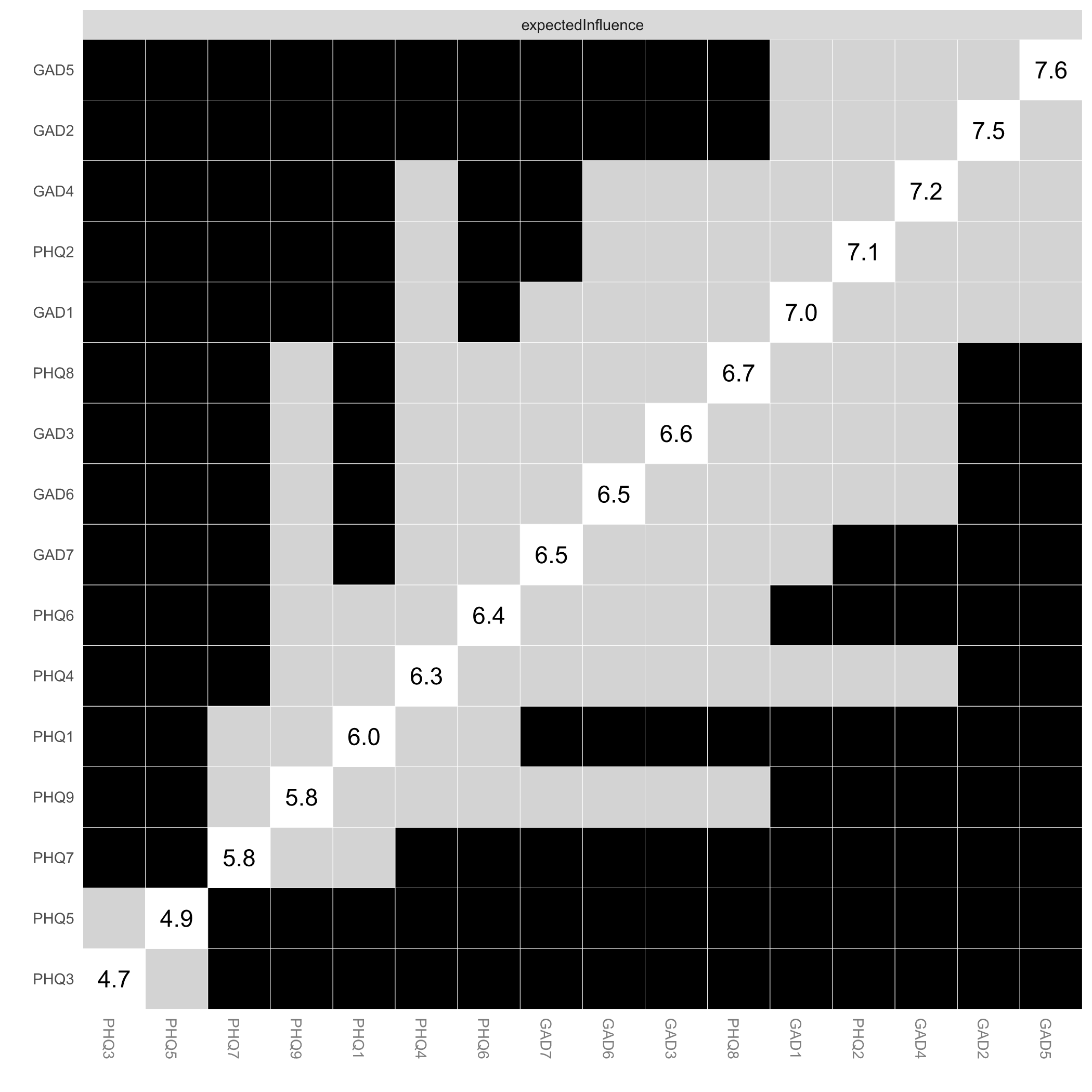

Supplement: Supplementary file 3 [file Image_2.TIFF]
